# Atherosclerosis and Malignant Disease

**DOI:** 10.1038/bjc.1965.2

**Published:** 1965-03

**Authors:** D. Eakins


### ATHEROSCLEROSIS AND MALIGNANT DISEASE

D. EAKINS

*From the Department of Pathology, Queen's University, Belfast*

Received for publication September 7, 1964

THERE are many studies of the association between atherosclerosis and malignant disease. Grosse (1958) in a review of the subject found that the cancer atherosclerosis relationship has been investigated by 15 authors in over 36,000 post mortem examinations, 11,000 of which were on cancer cases. He found that in all series the incidence and severity of atherosclerotic lesions are significantly less than in the controls.

The results of the large majority of these studies are based on the assessment of many observers using widely varying techniques. In the majority of series the cancer patients are separated from the remainder, thus selected groups of patients with malignant disease are compared with selected groups in whom coronary artery and cerebro-vascular disease probably form the principal causes of death.

The present investigation consists of a comparison of the extent and type of atherosclerosis occurring in patients with malignant disease and that found in an unselected autopsy series.

### MATERIALS AND METHODS

There were 141 patients whose ages ranged from the 4th to the 9th decade in whom a diagnosis of malignant disease was confirmed by histological examination of affected tissues. No other criteria were used for selection and the group, therefore, also includes patients who were obese, hypertensive or had ischaemic heart disease.

The extent and type of atherosclerosis in this group was compared with that in an unselected series of 595 subjects which includes the group of patients with malignant disease.

The aorta, coronary and cerebral vessels were examined after fixation in 10 per cent formol saline using the technique described by Gore and Tejada (1957) (Table I). The area in tenths of fatty, ulcerated and calcified lesions was measured separately and the remaining portion of tenths ascribed to fibrous lesions. In areas in which there was both ulceration and calcification each was measured separately. All the vessels were examined without knowledge of the sex, age or cause of death of the subjects. This information was subsequently obtained from the autopsy reports.

The observed distributions by the area or type of atherosclerosis, found at autopsy in subjects who had malignant disease, was compared with the distribution expected on the hypothesis that the proportionate distribution of area or type of atherosclerosis in these subjects is the same as that in all patients combined (i.e., null hypothesis).

Because of the differences in distribution of atherosclerosis between the two sexes these have been kept separate throughout the analysis. Both the observed and expected distributions have been standardised to allow for the possible effects of differences in the distribution by age of the various groups compared (Bradford Hill, 1961). The significance of the differences between these observed and expected distributions has been assessed by the chi squared test (Bradford Hill, 1961) and the level of significance used is $P > 0{\cdot}05$.

### RESULTS

Tables II and III show that in patients with malignant disease fewer specimens of aortic and coronary arteries fall into the larger atherosclerotic area groups (C, D) than expected on the basis of the distribution in the overall group. The differences are statistically significant in male patients in both aorta and coronary arteries and inspection of the data showed that these changes are consistent in all decades in both sexes.

Table IV shows that the observed numbers of cerebral artery specimens in the larger area groups is smaller than in the overall group, but the difference is not statistically significant.

Tables V, VI and VII show that no significant difference occurs between the observed numbers of patients with malignant disease of each sex with each proportion of atherosclerosis as fatty (Table V), ulcerative (Table VI), and calcified lesions (Table VII) and those expected on the basis of the distribution in the overall group after standardisation for age and area group. However, there is a proportionate decrease in the amount of calcified lesions in the aorta and coronary arteries of both sexes.

TABLE I.—*Area Groups of Atherosclerosis*

| Area Group | Proportion of intima affected with atherosclerosis |
|---|---|
| O . | Less than 5% |
| A . | 6–15% |
| B . | 16–33% |
| C . | 34–50% |
| D . | More than 50% |

TABLE II

Observed distribution of aortas in patients with malignant disease by sex and area group, compared within each sex, with that expected on the basis of the distribution in the overall group after standardisation for age.

| | Aorta | | | |
|---|---|---|---|---|
| | Male | | Female | |
| Area Group | Obs. | Exp. | Obs. | Exp. |
| O . | 0 | 0 | 2 | 1·2 |
| A . | 11 | 7·5 | 5 | 6·5 |
| B . | 11 | 5·9 | 4 | 3·9 |
| C . | 21 | 21·6 | 22 | 16·7 |
| D . | 23 | 31 | 17 | 21·7 |
| Total | 66 | 66 | 50 | 50 |
| $\chi^2$ . | 8·12 | | 2·45 | |
| D.F. . | 3 | | 2 | |
| P. . | 0·02–0·05 | | 0·2–0·3 | |

TABLE III

Observed distribution of coronary arteries in patients with malignant disease by sex and area group, compared within each sex, with that expected on the basis of the distribution in the overall group after standardisation for age.

| | Coronary arteries | | | |
|---|---|---|---|---|
| | Male | | Female | |
| Area Group | Obs. | Exp. | Obs. | Exp. |
| O | 7 | 2·8 | 8 | 4·35 |
| A | 24 | 16·2 | 13 | 12·5 |
| B | 22 | 11·5 | 9 | 7·3 |
| C | 18 | 21·3 | 12 | 15·0 |
| D | 6 | 25·2 | 13 | 15·9 |
| Total | 77 | 77 | 55 | 55 |
| $\chi^2$ | 32·31 | | 2·61 | |
| D.F. | 3 | | 3 | |
| P. | -0·001 | | 0·3–0·5 | |

TABLE IV

Observed distribution of cerebral arteries in patients with malignant disease by sex and area group, compared within each sex, with that expected on the basis of the distribution in the overall group after standardisation for age.

| | Cerebral arteries | | | |
|---|---|---|---|---|
| | Male | | Female | |
| Area Group | Obs. | Exp. | Obs. | Exp. |
| O | 24 | 20·7 | 19 | 17·0 |
| A | 15 | 18·0 | 13 | 11·4 |
| B | 11 | 6·1 | 3 | 2·8 |
| C | 8 | 7·5 | 5 | 6·6 |
| D | 2 | 7·7 | 4 | 6·2 |
| Total | 60 | 60 | 44 | 44 |
| $\chi^2$ | 9·30 | | 1·61 | |
| D.F. | 4 | | 3 | |
| P. | 0·05–0·1 | | 0·5–0·7 | |

### DISCUSSION

The results of this series are in general agreement with the majority of reports on the cancer atherosclerosis relationship published in the world literature. In details of the changes found they vary, however.

Rosenthal (1934) found a reduction in the total fat content of aortas of cancer patients when compared with a control group, while Wilens (1947) considered that the early stages of atherosclerosis could recede rapidly and also found less lipid in the intima of patients with weight loss than in the controls. Creed *et al.* (1955), however, considered that rapid weight loss may be associated with the development of atherosclerosis because of the overloading of the blood plasma with lipid from the fat stores.

In this present series there was no significant alteration in the proportion of fatty plaques in the atherosclerotic intima and it is of interest that 24 of the 141 patients were still obese at the time of autopsy. Resorption of fatty plaques alone

### Table V

Observed distribution of aortas and coronary arteries in patients with malignant disease, in area groups ABCD, by sex and proportion of atherosclerosis as fatty lesions, compared with that expected on the basis of the distribution in the overall group after standardisation for age and area group.

| Proportion of atherosclerosis as fatty lesions in tenths | Aorta | | | | Coronary arteries | | | |
|---|---|---|---|---|---|---|---|---|
| | Male | | Female | | Male | | Female | |
| | Obs. | Exp. | Obs. | Exp. | Obs. | Exp. | Obs. | Exp. |
| 0 | 0 | 0·3 | — | — | — | — | — | — |
| 1 | 6 | 4·3 | 4 | 3·7 | — | 0·1 | 2 | 1·0 |
| 2 | 10 | 11·2 | 5 | 5·9 | 1 | 2 | 1 | 0·6 |
| 3 | 6 | 7·7 | 7 | 6·3 | 1 | 1·9 | 1 | 4·0 |
| 4 | 14 | 9·6 | 9 | 9·1 | 9 | 6·5 | 0 | 4·8 |
| 5 | 11 | 12·6 | 13 | 11·9 | 14 | 11·2 | 20 | 10·6 |
| 6 | 1 | 3·8 | 0 | 1·7 | 5 | 5·9 | 4 | 4·7 |
| 7 | 6 | 3·2 | 1 | 0·7 | 2 | 2·6 | 1 | 1·9 |
| 8 | 3 | 4·2 | 3 | 3·7 | 19 | 15·44 | 7 | 8·0 |
| 9 | 9 | 8·2 | 5 | 4·5 | 16 | 23·0 | 10 | 9·6 |
| 10 | — | 0·9 | 1 | 0·5 | 3 | 1·4 | 1 | 1·8 |
| Totals | 66 | 66 | 48 | 48 | 70 | 70 | 47 | 47 |
| $\chi^2$ | 3·93 | | 1·14 | | 3·00 | | 12·81 | |
| D.F. | 5 | | 5 | | 4 | | 4 | |
| P. | 0·5–0·7 | | 0·95–0·98 | | 0·5–0·7 | | 0·01–0·02 | |

Although $\chi^2$ is statistically significant in the coronary arteries in female patients, there is no trend in the proportion of fatty lesions to indicate clinical significance.

### Table VI

Observed distribution of aortas and coronary arteries in patients with malignant disease in area groups ABCD, by sex, and proportion of atherosclerosis as ulcerative lesions compared with that expected on the basis of the distribution in the overall group, after standardisation for age and area group.

| Proportion of atherosclerosis as ulcerative lesions in tenths | Aorta | | | | Coronary arteries | | | |
|---|---|---|---|---|---|---|---|---|
| | Male | | Female | | Male | | Female | |
| | Obs. | Exp. | Obs. | Exp. | Obs. | Exp. | Obs. | Exp. |
| 0 | 28 | 28·6 | 26 | 24·5 | 56 | 52·4 | 35 | 30·0 |
| 1 | 28 | 28·8 | 20 | 19·8 | 10 | 14·1 | 11 | 12·7 |
| 2 | 6 | 6·0 | 1 | 3·2 | 3 | 2·9 | 1 | 3·6 |
| 3 | 3 | 2 | 1 | 0·5 | 1 | 0·4 | — | 0·7 |
| 4 | 1 | 0·4 | — | 0·1 | — | 0·2 | — | — |
| 5 | — | — | — | — | — | — | — | — |
| 6 | — | 0·1 | — | — | — | — | — | — |
| 7 | — | 0·1 | — | — | — | — | — | — |
| 8 | — | — | — | — | — | — | — | — |
| 9 | — | — | — | — | — | — | — | — |
| 10 | — | — | — | — | — | — | — | — |
| Totals | 66 | 66 | 48 | 48 | 70 | 70 | 47 | 47 |
| $\chi^2$ | 0·26 | | 0·20 | | 0·99 | | 2·30 | |
| D.F. | 2 | | 2 | | 1 | | 1 | |
| P. | 0·8–0·9 | | 0·9–0·95 | | 0·3–0·5 | | 0·1–0·2 | |

TABLE VII

Observed distribution of aortas and coronary arteries in patients with malignant disease in area groups ABCD, by sex and proportion of atherosclerosis as calcified lesions, compared with that expected on the basis of the distribution in the overall group, after standardisation for age, and area group.

| Proportion of atherosclerosis as calcified lesions in tenths | Aorta | | | | Coronary arteries | | | |
|---|---|---|---|---|---|---|---|---|
| | Male | | Female | | Male | | Female | |
| | Obs. | Exp. | Obs. | Exp. | Obs. | Exp. | Obs. | Exp. |
| 0 | 39 | 37·8 | 29 | 28·0 | 60 | 56·7 | 31 | 30·5 |
| 1 | 20 | 22·9 | 10 | 11·0 | 9 | 12·2 | 14 | 12·3 |
| 2 | 7 | 4·9 | 7 | 7·0 | 1 | 0·8 | — | 3·1 |
| 3 | — | 0·4 | 1 | 2·0 | — | 0·3 | 1 | 0·6 |
| 4 | — | — | 1 | 0 | — | — | 1 | 0·5 |
| 5 | — | — | — | — | — | — | — | — |
| 6 | — | — | — | — | — | — | — | — |
| 7 | — | — | — | — | — | — | — | — |
| 8 | — | — | — | — | — | — | — | — |
| 9 | — | — | — | — | — | — | — | — |
| 10 | — | — | — | — | — | — | — | — |
| Totals | 66 | 66 | 48 | 48 | 70 | 70 | 47 | 47 |
| | 0·96 | | 0·13 | | 1·01 | | 0·03 | |
| D.F. | 2 | | 2 | | 1 | | 1 | |
| P. | 0·10–0·21 | | 0·9–0·95 | | 0·2–0·3 | | 0·8–0·9 | |

does not, therefore, account for the reduction in extent of atherosclerosis in these cancer patients.

Results of a comparison between those subjects who were thin and emaciated at autopsy and the overall group and this present group and the overall group appears to indicate that the reduction in severity of atherosclerosis is more marked in those patients with malignant disease (Eakins, 1963). This would indicate that weight loss alone does not account for the reduction of atherosclerosis in malignant disease.

Hueck (1920) considered that cachexia from cancer favours calcification of arteries. Elkeles (1959), however, found that patients with cancer (except of the respiratory tract) had much less calcification in their abdominal aortas on radiological examination than had controls. He suggested that a biochemical system with an increased affinity of the lipoids and proteins to calcium leads to calcified atherosclerosis and to relative immunity to cancer.

This present study does not substantiate any significant alteration in the proportion of calcification of atherosclerotic aortic or coronary artery intima. There is, however, a small but consistent increase in both sexes in those without calcification (Table VI). This small reduction does not, however, appear to substantiate the findings of Elkeles (1959).

It has been shown that as the area of intima involved with atherosclerosis increases so also does the proportion of that intima which is calcified or ulcerated (Eakins 1963). A group of patients, therefore, with a lower extent of atherosclerosis than the overall group will also have a smaller proportion of calcified and ulcerative lesions. The reduction in the extent and severity of the atherosclerosis both as regards ulcerative and calcified lesions may account for Elkeles' (1959) radiological findings rather than a lack of affinity of the fat and protein in the atherosclerotic plaques for calcium salts. Juhl (1955) found atherosclerotic lesions less pronounced in cancer patients than in controls and considered that tumours inhibit in some way the development of atherosclerosis and that the basis of this inhibition should be sought in the relationship of cholesterol or its derivatives to the development and growth of malignant tumours. This has, however, not been substantiated.

The attempts, therefore, to explain the reduced severity of atherosclerosis in malignant disease on a basis of weight loss or hypotheses on biochemical systems with an affinity for calcium are not supported by this survey.

The distribution of malignant growths, e.g., of the stomach (Macafee, 1962) and ischaemic heart disease (Brönte Stewart, 1961) between different blood groups and the reported familial incidence of hypertension and hypercholesterolaemia (Boas *et al.*, 1948) raise the possibility of a genetic mechanism being the basis for the reduced severity of atherosclerosis in these patients. However, this is almost entirely speculative and no clear answer is available at present for the findings in this survey.

### SUMMARY

A macroscopic study of atherosclerosis in 140 patients with malignant disease reveals a reduction in extent of intima affected but no alteration in the proportionate distribution of fatty ulcerative and calcified lesions.

### REFERENCES

Boas, E. P., Parets, A. D. and Adlersberg, D.—(1948) *Amer. Heart J.*, **35**, 611.
Bradford-Hill, A.—(1961) 'Principle of Medical Statistics'. 7th Edition. London (The Lancet) Ltd., p. 202.
Brönte Stewart, B.—(1961) 'Recent Advances in Human Nutrition'. Edited by J. F. Brock. London (J. and A. Churchill Ltd.), p. 183.
Creed, D. I., Baird, W. F. and Fischer, E. R.—(1955) *Amer. J. med. Sci.*, **230**, 385.
Eakins, D.—(1963) M.D. Thesis 'Studies in atherosclerosis', Queen's University, Belfast.
Elkeles, A.—(1959) *Brit. J. Cancer*, **13**, 403.
Gore, I. and Tejada, C.—(1957) *Amer. J. Path.*, **33**, 857.
Grosse, H.—(1958) *Z. Krebsforsch.*, **65**, 519.
Hueck.—(1920) cited by Juhl, S. (1955).
Juhl, S.—(1955) *Acta path. microbiol. scand.*, **37**, 167.
Macafee, A. L.—(1962) M.D. Thesis 'Blood groups and disease'. Queen's University, Belfast.
Rosenthal, S. R.—(1934) *Arch. Path.*, **18**, 473.
Wilens, S. L.—(1947) *Amer. J. Path.*, **23**, 793.


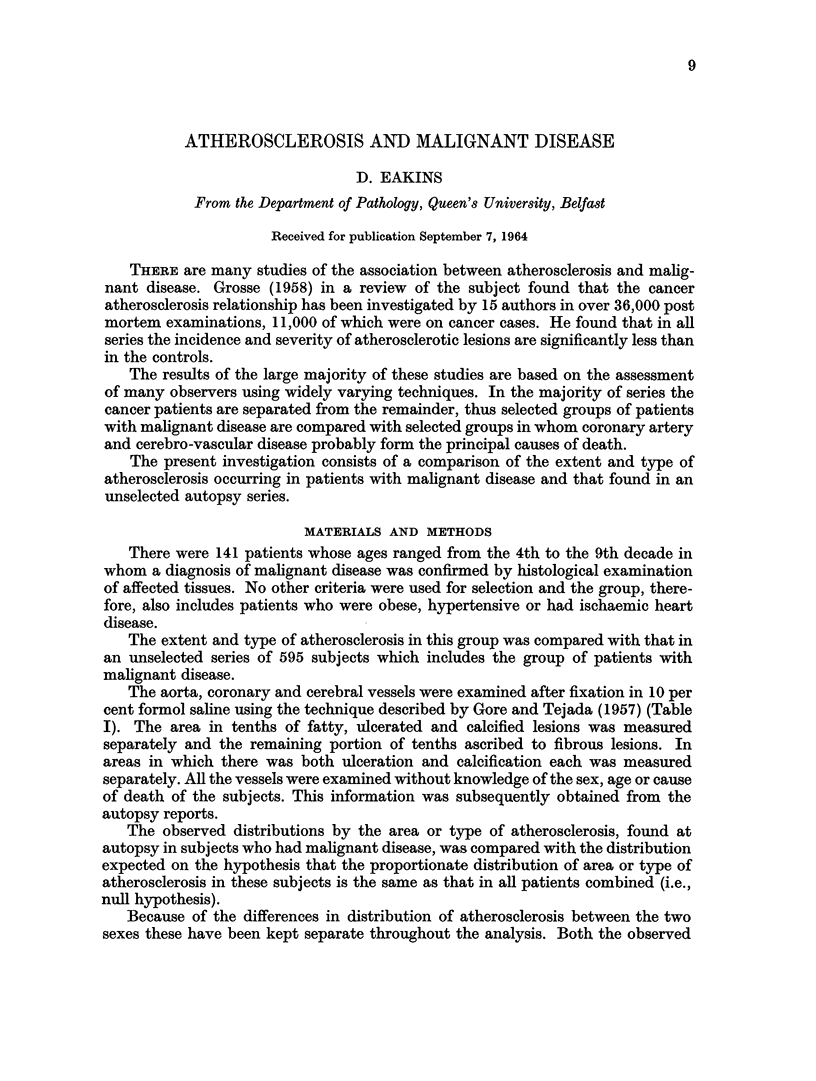

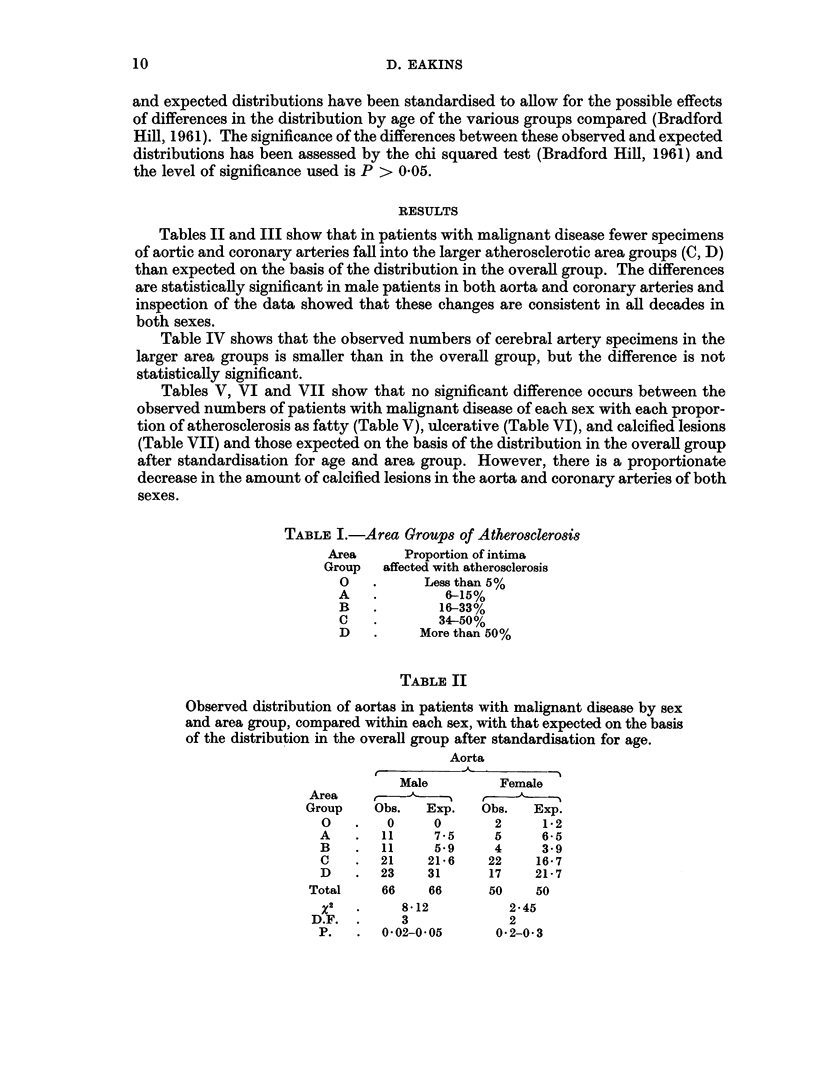

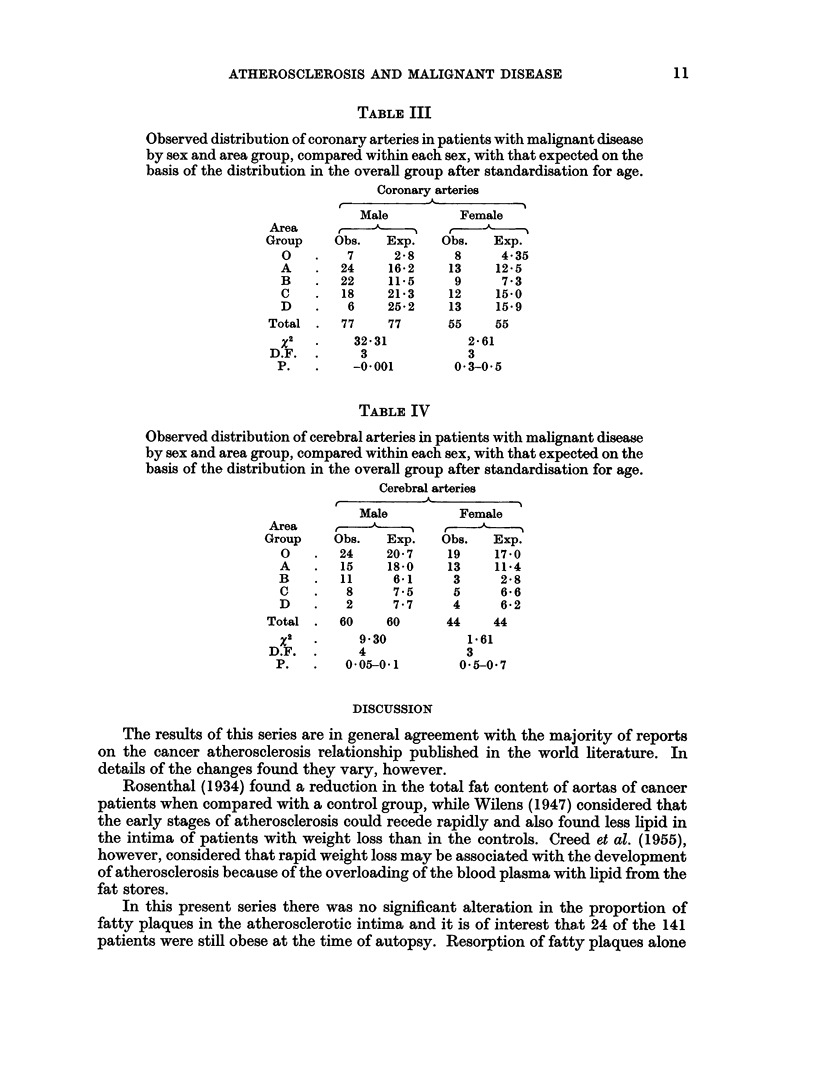

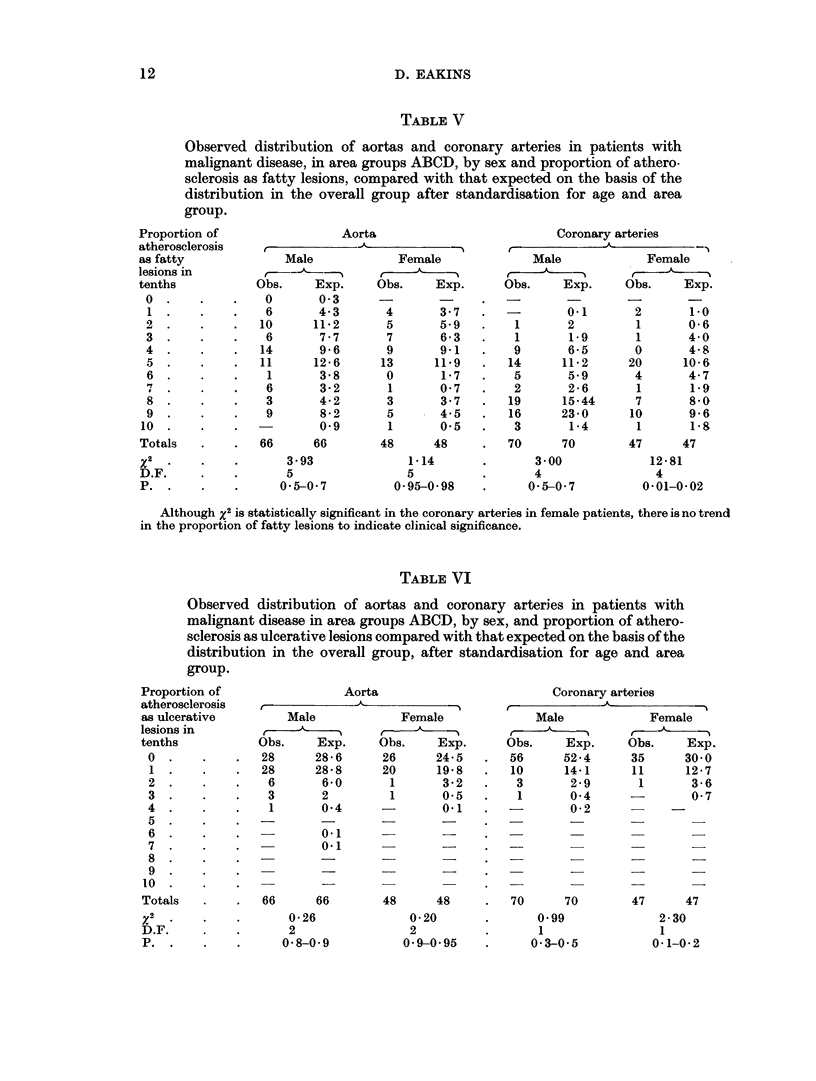

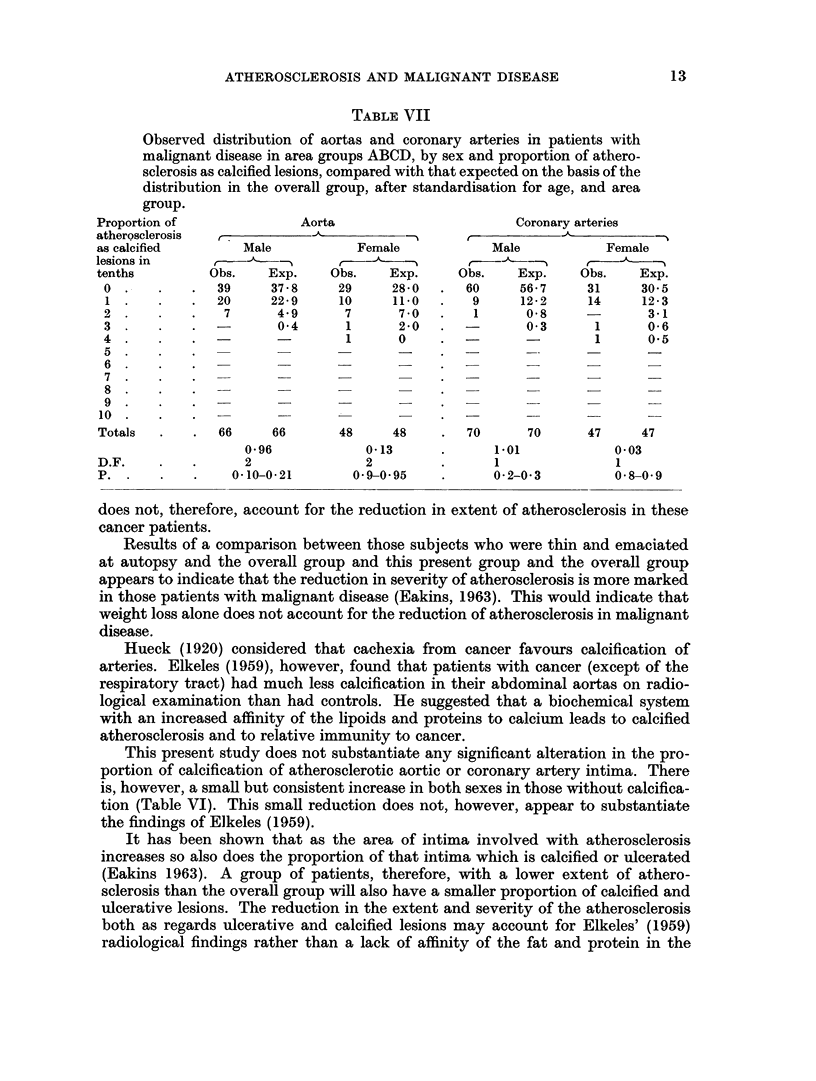

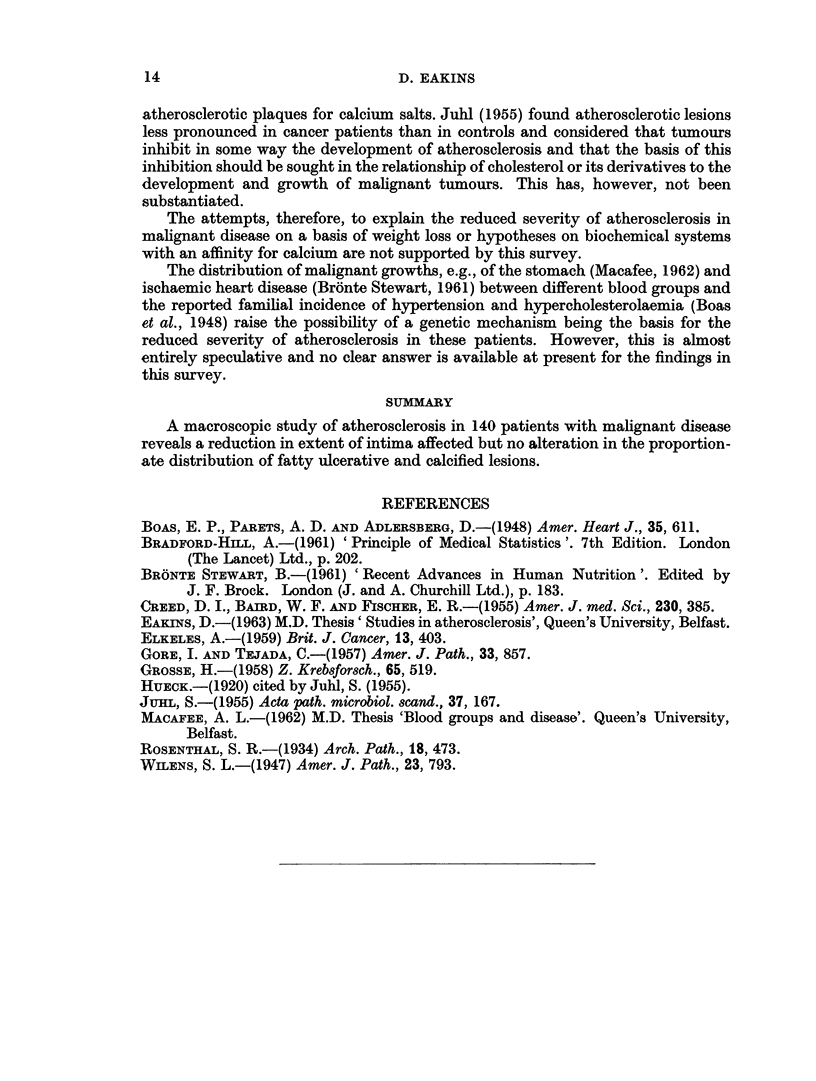


## References

[OCR_00616] CREED D. L., BAIRD W. F., FISHER E. R. (1955). The severity of aortic arteriosclerosis in certain diseases; a necropsy study.. Am J Med Sci.

[OCR_00617] ELKELES A. (1959). Calcified atherosclerosis and cancer.. Br J Cancer.

[OCR_00620] GROSSE H. (1958). Arteriosklerose und Krebs.. Z Krebsforsch.

[OCR_00621] JUHL S. (1955). Cancer and atherosclerosis: negative correlation.. Acta Pathol Microbiol Scand.

[OCR_00630] Wilens S. L. (1947). The Resorption of Arterial Atheromatous Deposits in Wasting Disease.. Am J Pathol.

